# Adverse events following immunization and psychological distress among cancer patients/survivors following vaccination against SARS-CoV-2 infection

**DOI:** 10.3389/fpsyg.2022.906067

**Published:** 2022-07-26

**Authors:** Li Ping Wong, Lee Lee Lai, Mee Hoong See, Haridah Alias, Sharifah Faridah Syed Omar, Chong Guan Ng, Gwo Fuang Ho, Teng Aik Ong, Yee Chi Wong, Po Lin Ooi, Jasmin Munchar Elias, Zhijian Hu, Yulan Lin

**Affiliations:** ^1^Centre for Epidemiology and Evidence-Based Practice, Department of Social and Preventive Medicine, Faculty of Medicine, Universiti Malaya, Kuala Lumpur, Malaysia; ^2^Department of Epidemiology and Health Statistics, School of Public Health, Fujian Medical University, Fuzhou, China; ^3^Department of Nursing Science, Faculty of Medicine, Universiti Malaya, Kuala Lumpur, Malaysia; ^4^Breast Surgery Unit, Department of Surgery, Faculty of Medicine, Universiti Malaya, Kuala Lumpur, Malaysia; ^5^Infectious Disease Unit, Department of Medicine, Universiti Malaya, Kuala Lumpur, Malaysia; ^6^Department of Psychological Medicine, Faculty of Medicine, Universiti Malaya, Kuala Lumpur, Malaysia; ^7^Clinical Oncology Unit, Faculty of Medicine, Universiti Malaya, Kuala Lumpur, Malaysia

**Keywords:** adverse events, cancer survivors, COVID-19 vaccine, anxiety, depression

## Abstract

**Purpose:**

This study aims to describe the adverse events following immunization (AEFIs) of SARS-CoV-2 vaccination in cancer patients/survivors associated with their psychological distress.

**Methods:**

A cross-sectional study was conducted to assess AEFIs after the receipt of SARS-CoV-2 vaccines in cancer patients/survivors attending a university hospital in Malaysia. Psychological distress was measured using the Hospital Anxiety and Depression Scale (HADS) before and after the first and second doses of COVID-19 vaccine.

**Results:**

A total of 217 complete responses were received. Compared with before vaccination, both HADS Anxiety (HADS-A) and HADS Depression (HADS-D) scores were significantly reduced after the first and second dose of the SARS-CoV-2 vaccine. Most of the participants had mild-or-moderate systemic and local AEFIs, with the most common being pain at the injection site, tiredness, and headache for both the first and second doses of the vaccine. Positive correlations between the total AEFI score and HADS-A (*r* = 0.309, *p* < 0.001) and HADS-D (*r* = 0.214, *p* = 0.001) scores were observed after the first dose of the SARS-CoV-2 vaccine. Similarly, positive associations were observed between the total AEFI score and HADS-A (*r* = 0.305, *p* < 0.001) and HADS-D (*r* = 0.235, *p* < 0.001) scores after the second dose of the SARS-CoV-2 vaccine.

**Conclusion:**

Mild-to-moderate AEFIs found in this study help address vaccine hesitancy in cancer patients/survivors. Receiving the SARS-CoV-2 vaccine had a positive effect on decreasing psychological distress in cancer patients/survivors. High severity of an AEFI was associated with higher anxiety and depressive symptoms.

## Introduction

Since the onset of the global pandemic of coronavirus disease-2019 (COVID-19), the highly contagious viral illness caused by SARS-CoV-2, the lives of cancer patients/survivors have dramatically changed. It has been well established that the high-risk populations that may be threatened by SARS-CoV-2 infection are cancer patients. Cancer patients have an elevated susceptibility to severe COVID-19 disease that can be attributed to the immunosuppressed status caused by the disease itself or anticancer treatments, such as chemotherapy or surgery (Al-Quteimat and Amer, 2020). Cumulated evidence has shown that cancer patients not only have a high risk of serious complications from COVID-19 but also have an increased risk for COVID-19-related death (ElGohary et al., 2020; Gupta et al., 2021; Russell et al., 2021). Considering the increased vulnerability of cancer patients, it is well known that pandemics pose a threat to the mental health of this population. Fear of infection, lockdowns, social distancing, and curfews have severe impacts on mental cancer patients/survivors and worsen their condition. A global systematic review revealed that COVID-19 greatly affects the psychological health of cancer patients (Momenimovahed et al., 2021). A local study likewise revealed that Malaysian cancer patients/survivors experienced high rates of emotional distress during the COVID-19 pandemic (Wong et al., 2021).

The vaccines against SARS-CoV-2 were developed at an unprecedented rate and have been an extraordinary success (Ball, 2021). Approximately 1 year into the COVID-19 pandemic, vaccines against SARS-CoV-2 have been authorized for emergency use by the U.S. Food and Drug Administration (FDA; FDA, 2022). After their FDA approval, the Advisory Committee on Immunization Practices (ACIP) recommended that healthcare personnel and long-term care facility residents be offered COVID-19 vaccination first (Dooling, 2021). Malaysia launched its COVID-19 immunization plan on 24 February 2021 (Ministry of Health Malaysia, 2021a). Frontline workers, the elderly, and vulnerable populations, such as the elderly and people with chronic illnesses (including cancer patients/survivors), were the first phase priority groups for SARS-CoV-2 vaccination (Ministry of Health Malaysia, 2021b). However, there remain many uncertainties in safety, efficacy, and adverse events following immunization (AEFIs) in the frail population, particularly in light of the lack of representation or exclusion of patients with cancer from the pivotal clinical trials of SARS-CoV-2 vaccines (Buttiron Webber et al., 2021; Trapani and Curigliano, 2021). As cancer patients/survivors are frailer compared to healthy people, all health events after vaccination against SARS-CoV-2 among cancer patients/survivors are important to investigate. An understanding of AEFIs and background and clinical characteristics of cancer patients/survivors who have had other serious AEFIs may provide insights into targeted patient groups that need close monitoring and medical supervision following vaccination.

Little is known about the acceptability of the SARS-CoV-2 vaccine in people with health conditions. A study found that individuals with serious comorbid conditions exhibit significant vaccine hesitancy and doubts about vaccine safety (Tsai et al., 2022), particularly after reports of thrombosis and coagulation abnormalities following SARS-CoV-2 vaccination (Cari et al., 2021). Lack of empirical evidence of the AEFIs of SARS-CoV-2 vaccination in cancer patients/survivors may lead to vaccine hesitancy and instill fear and anxiety of vaccination. Much research has investigated the psychological wellbeing of cancer patients/survivors during the COVID-19 pandemic. However, relatively little is known about the psychological distress surrounding cancer patients/survivors at the time of receiving the SARS-CoV-2 vaccine and after receiving the vaccine. Cancer patients/survivors in Malaysia are the priority group in the SARS-CoV-2 vaccine rollout. Being the first among the general public to receive the vaccine may raise concerns about its newness, safety, and potential side effects. On the other hand, the rollout of the SARS-CoV-2 vaccines in Malaysia began during the period of rapid escalation of cases in the country. It is unsure if receiving the SARS-CoV-2 vaccine may provide a sigh of relief for cancer patients during the pandemic.

To date, there has been relatively little research comparing the psychological distress of cancer patients/survivors before and after SARS-CoV-2 vaccination. It is unknown whether receiving SARS-CoV-2 vaccination generates or eases psychological distress in cancer patients is unknown. Furthermore, it is also unknown if there is any association between the experience of AEFIs of SARS-CoV-2 vaccination and psychological distress among cancer patients/survivors. To shed light on this knowledge gap, this study aimed to better understand the AEFIs of SARS-CoV-2 vaccination in cancer patients/survivors and to associate the AEFIs with psychological wellbeing during the COVID-19 pandemic.

## Materials and methods

### Study participants and study design

**Figure 1:**
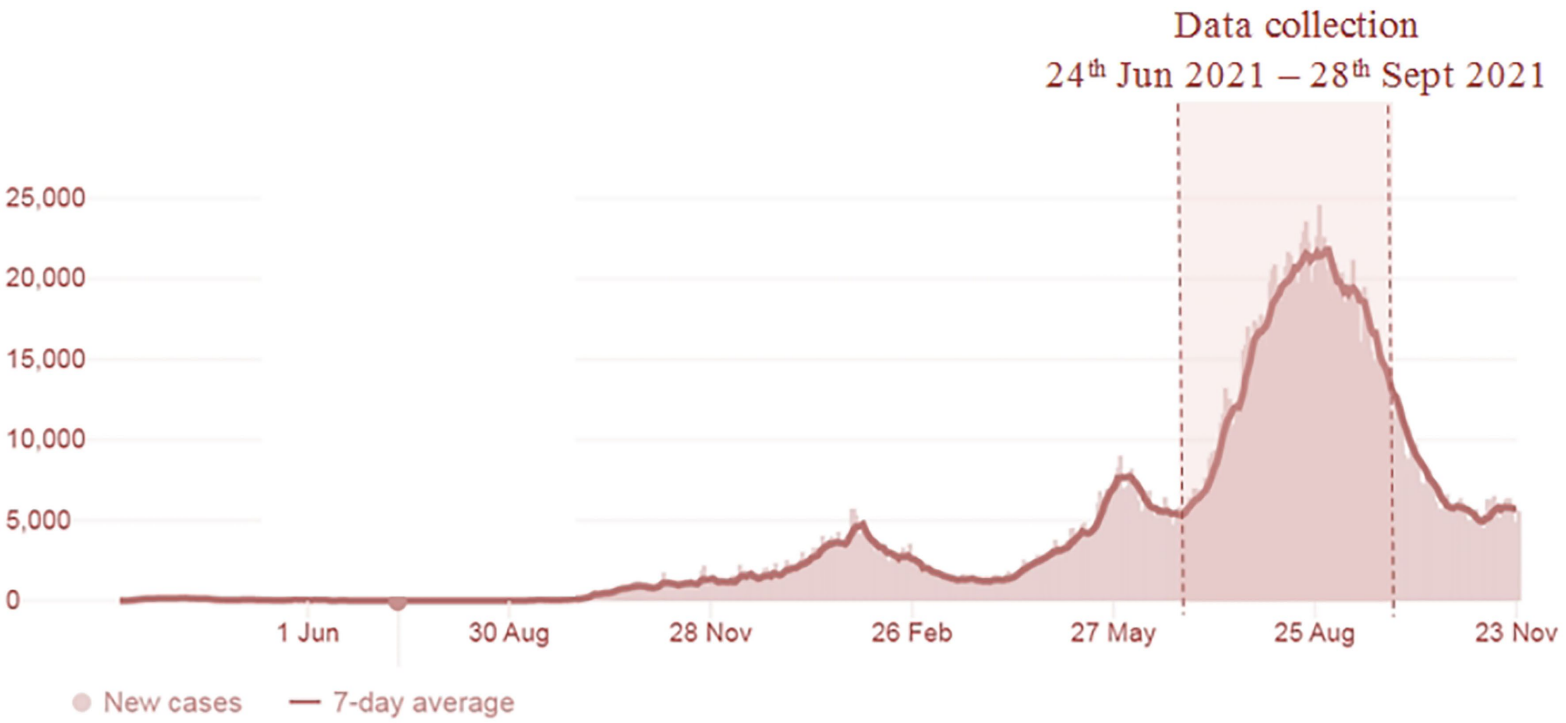
Data collection period and the trend of COVID-19 cases in Malaysia.

The study was conducted between 24 June 2021 and 29 August 2021. The data collection period, with the trend of the confirmed COVID-19 cases in Malaysia, is shown in Figure 1. Study participants were cancer patients/survivors from the Universiti Malaya Medical Centre (UMMC) scheduled to receive the SARS-CoV-2 vaccine from its COVID-19 Vaccination Program. Inclusion criteria for this study were as follows: (1) being older than 18 years of age; (2) having a diagnosis of at least 2 years; (3) currently not receiving treatment, except for endocrine therapy; (4) being a Malaysian citizen; and (5) receiving SARS-CoV-2 vaccine appointment confirmation from the UMMC. Participants were invited to complete three survey questionnaires (Appendix 1): (1) before the administration of the first dose of the SARS-CoV-2 vaccine, (2) 1 week after administration of the first dose of the SARS-CoV-2 vaccine; and (3) 1 week after administration of the second dose of the SARS-CoV-2 vaccine. All respondents were informed that their participation was voluntary. As the study was carried out during the pandemic, Google Forms was used to set up the survey online. The survey link was sent to patients’ mobile, and they answered the questions guided by the researcher. Informed consent was obtained using an online consent form. To consent to participate, participants were required to click “Yes, I consented to participate in this study.”

### Instruments

#### Pre-vaccination survey questions

The pre-vaccination survey consisted of sections of questions that assessed: (1) sociodemographic background, characteristics of cancer, and perceived current health status and (2) psychological distress measured using the Hospital Anxiety and Depression Scale (HADS).

Psychological distress was measured using the HADS (Zigmond and Snaith, 1983). The HADS is a valid and reliable self-rating scale that measures anxiety and depression in both hospitals and communities and is the most extensively validated scale for screening emotional distress in cancer patients (Vodermaier and Millman, 2011). The Bahasa Malaysia version of the HADS questionnaire was used (Yahya and Othman, 2015). The anxiety subscale of the Bahasa Malaysia version of the HADS questionnaire demonstrated a sensitivity of 90.0% and specificity of 86.2%, whereas the depression subscale demonstrated a sensitivity of 93.2% and specificity of 90.8% (Yahya and Othman, 2015).

#### Post-vaccination survey questions

The post-vaccination survey consisted of sections of questions that assessed: (1) psychological distress measured using the HADS and (2) AEFIs of SARS-CoV-2 vaccination. The post-vaccination survey was disseminated 1 week after the date of the first dose and the second dose of SARS-CoV-2 vaccine administration, respectively. The AEFI items comprised questions on 10 adverse events and a provision of free-text reporting for any other adverse events. The AEFIs of SARS-CoV-2 vaccination symptom experience was assessed by a list of the 10 most commonly reported side effects (CDC, 2021), namely tenderness at the injection site, headache, chills, joint pain, fever, nausea, feeling unwell, swelling of the lymph nodes, and thrombotic events. The option responses for each side effect were “none,” “mild,” “moderate,” and “severe.” Individual scale items are scored on a 4-point continuum (0 = none, 3 = severe). Item scores were summed to form a total score. Higher scores indicate greater levels of AEFIs regarding SARS-CoV-2 vaccination.

The questionnaire is available in two different languages, English and Bahasa Malaysia, (the native language in Malaysia). The questionnaire was pilot tested in 20 cancer patients/survivors recruited from the oncology and breast surgery outpatient clinics in UMMC. The average time taken to answer all the questions was 10 min.

### Ethical considerations

The study was approved by the UMMC Medical Research Ethics Committee (MREC) (Approval code: MREC ID No: 202166-10200). This study was conducted in accordance with the principles of the Declaration of Helsinki. The study participants were informed as to the purpose of the study, and that informed consent included consent to have anonymized responses published. It was also noted in the survey form that consent was implied upon completion of the questionnaire. All responses were collected and analyzed without identifiers. A note (with contact information) indicating the availability of counseling services for participants experiencing psychological distress was also included in the participant’s information sheet.

### Statistical analysis

An analysis of descriptive statistics was conducted to illustrate the demographics, scores on HADS, and total AEFIs of COVID-19 vaccination. Wilcoxon Signed-Rank Test was used to compare the scores on HADS and total AEFIs of COVID-19 vaccination before and after vaccination. Spearman’s correlation coefficient (r) was used to evaluate the correlation between the total AEFIs of COVID-19 vaccination and the HADS-A and HADS-D scores. Statistically significant variables were screened and included in multivariate logistic regression analyses. Multivariable logistic regression analyses were carried out only if more than two significant factors were found in univariate analyses. The estimates of the strengths of associations were demonstrated by odds ratios (ORs) with 95% confidence intervals (CIs). Hosmer-Lemeshow goodness-of-fit tests were used to ensure that the models adequately fit the data. A two-tailed *p* < 0.05 was considered statistically significant. All analyses were also conducted using SPSS version 22.0 (SPSS Inc., Chicago, IL, United States).

## Results

A total of 217 complete responses from cancer patients/survivors were received between 24 June 2021 and 28 September 2021. Figure 1 shows the study duration and the COVID-19 cases in Malaysia. The survey was carried out during the escalating increase of COVID-19 cases in the country. As shown in Table 1, the majority of the study participants are females (84.3%) of Chinese ethnicity (71.4%). A total of 70.0% of study participants were college or university graduates and slightly over half (52.5%) had incomes of MYR5000 and below. Most of the study participants had been diagnosed with cancer for 1–5 years (69.1%). Only 20 participants (9.2%) were diagnosed with more than one type of cancer. The majority were in stage 1 (32.3%) and stage 1 (26.3%) of cancer at the time of the first diagnosis. Only 13.8% of participants reported having been diagnosed with other comorbidities, and slightly over one-third (37.3%) of participants reported that their health status was fair/poor.

**Table 1:** Demographics of study participants and baseline of the Hospital Anxiety and Depression Scale (HADS; *N* = 217).

Socio-demography		Univariable analysis		Multivariable analysis	Univariable analysis	
		HADS-Anxiety (>8)	Value of *p*	OR (95 CI%; >8 vs ≤ 8)	HADS- Depression (>8)	Value of *p*
Age group (years)						
19–40	52 (24.0)	15 (28.8)	0.944		5 (9.6)	
41–50	81 (37.3)	22 (27.2)			10 (12.3)	0.117
60–81	84 (38.7)	22 (26.2)			18 (21.4)	
Gender						
Male	34 (15.7)	9 (26.5)	1.000		6 (17.6)	0.612
Female	183 (84.3)	50 (27.3)			27 (14.8)	
Ethnicity						
Malay	40 (18.4)	9 (22.5)	0.682		6 (15.0)	0.606
Chinese	155 (71.4)	42 (27.1)			22 (14.2)	
Indian	12 (5.5)	4 (33.3)			2 (16.7)	
Others	10 (4.6)	4 (40.0)			3 (30.0)	
Religion						
Islam	43 (19.8)	9 (20.9)	0.769		6 (14.0)	0.833
Buddhism	111 (51.2)	31 (27.9)			19 (17.1)	
Hinduism	11 (5.1)	4 (36.4)			2 (18.2)	
Christianity	41 (18.9)	11 (26.8)			4 (9.8)	
Others	11 (5.1)	4 (36.4)			2 (18.2)	
Highest educational level					
Secondary and below	65 (30.0)	24 (36.9)	0.045	1.73 (0.89–3.36)	13 (20.0)	0.218
Tertiary	152 (70.0)	35 (23.0)		Reference	20 (13.2)	
Average monthly household income (MYR)				
≤5,000	114 (52.5)	36 (31.6)	0.311		20 (17.5)	0.206
5,001–10,000	67 (30.9)	15 (22.4)			11 (16.4)	
>10,000	36 (16.6)	8 (22.2)			2 (5.6)	
*Cancer characteristics*						
Number of cancer diagnosed with				
1	197 (90.8)	50 (25.4)	0.069		31 (15.7)	0.745
>1	20 (9.2)	9 (45.0)			2 (10.0)	
Duration of being diagnosed with cancer (years)				
Less than 1	15 (6.9)	5 (33.3)	0.317		3 (20.0)	0.295
1–5	150 (69.1)	44 (29.3)			19 (12.7)	
>5	52 (24.0)	10 (19.2)			11 (21.2)	
Stage of cancer in time of diagnosis^†^				
0	25 (11.5)	9 (36.0)	0.696		2 (8.0)	0.616
1	57 (26.3)	16 (28.1)			12 (21.1)	
2	70 (32.3)	20 (28.6)			10 (14.3)	
3	44 (20.3)	10 (22.7)			6 (13.6)	
4	21 (9.7)	4 (19.0)			3 (14.3)	
*Current health condition*				
Diagnosed with other comorbidities					
Yes	30 (13.8)	14 (46.7)	0.014	2.26 (0.98–5.21)	5 (16.7)	0.787
No	187 (86.2)	45 (24.1)		Reference	28 (15.0)	
Perceived current health status				
Very good/Good	136 (62.7)	24 (17.6)	*p* < 0.001	Reference	14 (10.3)	0.011
Fair/Poor	81 (37.3)	35 (43.2)		3.23 (1.71–6.10)^**^	19 (23.5)	

^**^p < 0.01.

a Hosmer–Lemeshow test, Chi-square: 11.86, value of *p*: 0.018; Nagelkerke *R*^2^: 0.146.

† Level 0 denotes ductus carcinoma in sit.

### Hospital anxiety and depression scale

The baseline mean HADS-A and HADS-D scores for the study participants were 6.0 (±3.9) and 4.2 (±3.6), respectively. In total, before receiving the vaccine, 27.2% (95% CI 21.4–33.6) of participants reported a HADS-A score >8 and 15.2% (95% CI 10.7–20.7) had a HADS-D score >8. The multivariable analysis shown in Table 1 indicates that HADS-A score >8 was significantly higher in participants who perceived their current health status as fair/poor (aOR: 3.2; 95% CI: 1.7–6.1). The association between participants diagnosed with other comorbidities and HADS-A scores >8 (aOR 2.3; 95% CI 1.0–5.2) was not statistically significant. Perceived current health status was the only significant factor associated with HADS-D scores >8 in the univariate analysis (OR: 2.7; 95% CI: 1.3-5.7).

**Figure 2:**
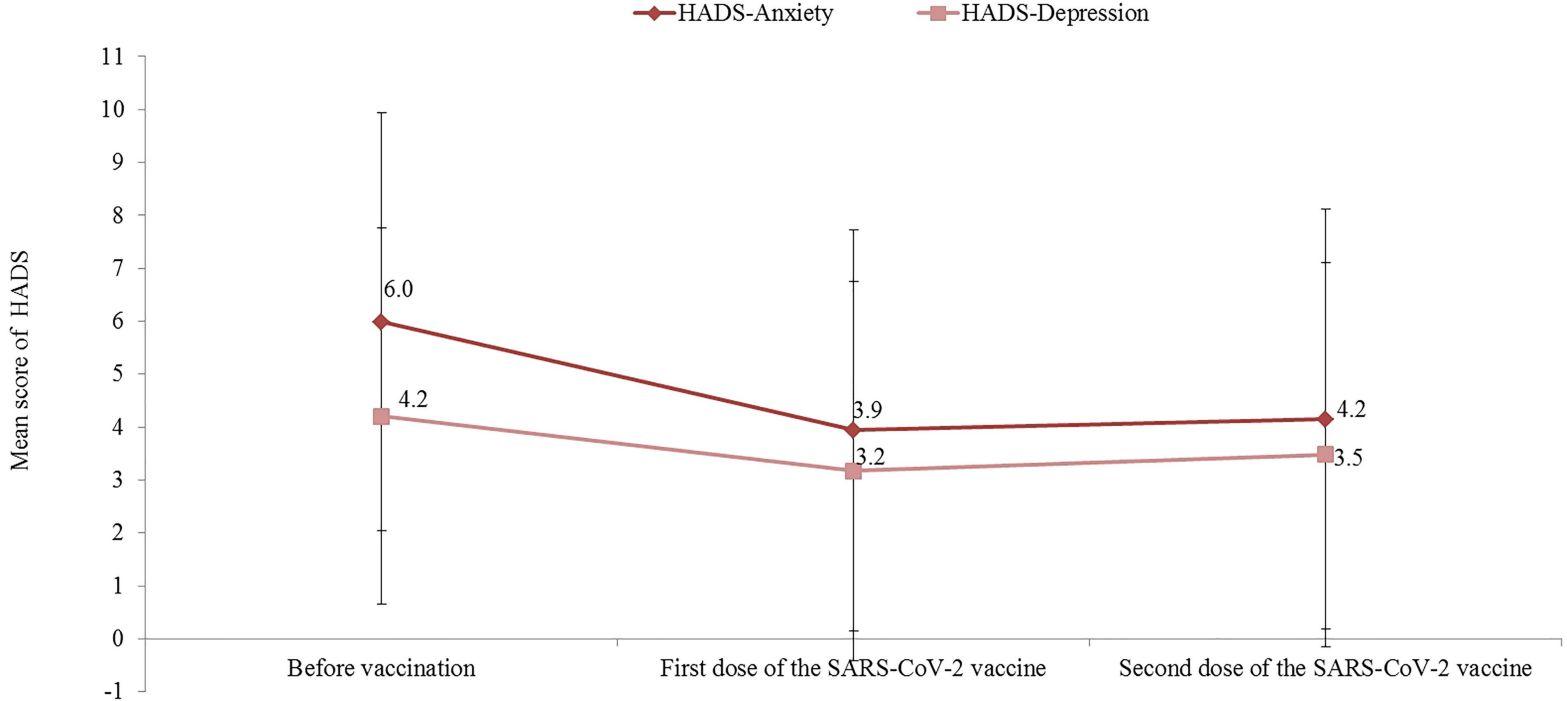
HADS-A and HADS-D before and after first and second doses of the SARS-CoV-2 vaccine.

Figure 2 shows the trends of the HADS-A and HADS-D scores during the study period. Compared with baseline values, both the HADS-A and HADS-D scores were significantly reduced after the first dose of the SARS-CoV-2 vaccine, with a mean difference of 2.1 (95% CI: mean difference: 1.6–2.4; *p* < 0.001) and 1.0 (95% CI mean difference: 0.7–1.4; *p* < 0.001), respectively. Similarly, compared with baseline values, a significant reduction between the baseline and HADS-A (mean difference = 1.8; 95% CI 1.4–2.3; *p* < 0.001) and HADS-D (mean difference = 0.7; 95% CI 0.3–1.2; *p* < 0.001) scores were observed after the second dose of the COVID-19 vaccine. In total, after receiving the second dose of the SARS-CoV-2 vaccine, only 13.8% (95% CI 9.5–19.1) of participants reported a HADS-A score >8 and 10.6% (95% CI 6.8–15.5) had a HADS-D score >8.

### AEFIs after administration of the first and second dose of the SARS-CoV-2 vaccine

**Figure 3:**
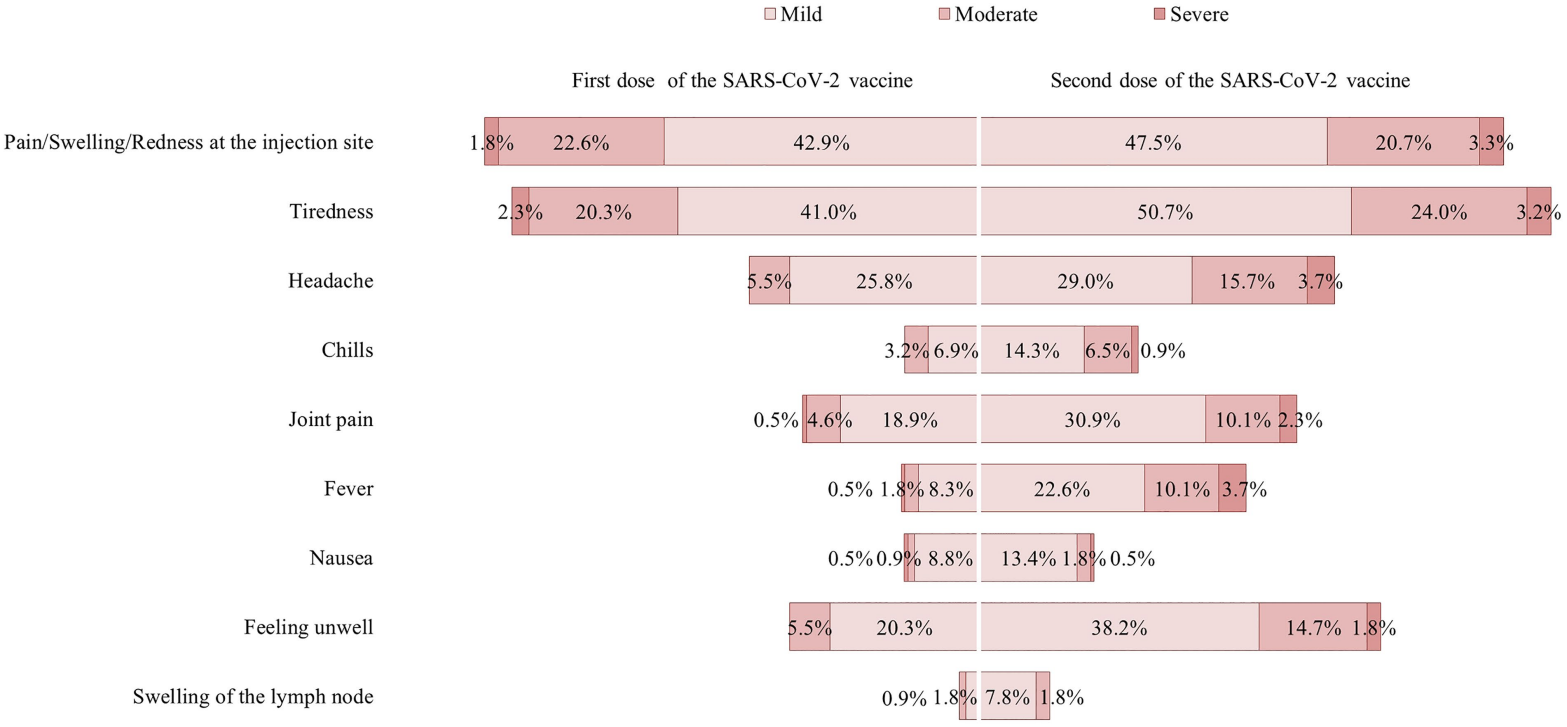
Level of severity of side effects after first and second doses of the SARS-CoV-2 vaccine.

The distributions of AEFIs after administration of the first and second dose of the SARS-CoV-2 vaccine are illustrated in Figure 3. Most of the AEFIs were mild-to-moderate, with the most common being pain at the injection site, tiredness, and headache for both the first dose and second dose of vaccine, respectively. There were no serious events requiring hospitalization among the study participants. Figure 3 also shows that the AEFIs were relatively higher after the second dose of vaccine when compared to that after the first dose of vaccine. When the AEFIs were summed, the mean AEFI score after the second dose of vaccine (5.2 ± 3.9) was significantly higher compared to that after the first dose of vaccine (3.2 ± 2.9; *p* < 0.001). The range of AEFI scores after the second dose of vaccine (0–20) was also higher when compared to that after the first dose of vaccine (0–16). The median and IQR for the AEFI score after the first dose of vaccine and the second dose of vaccine were 2 (IQR 1.0–5.0) and 3 (IQR 2.0–7.5), respectively. The AEFI scores were categorized as high or low based on the median split. Based on the median split, a total of 102 (47.0%) participants were categorized as having a high AEFI score (3–16) and 115 (53.0%) as having a low AEFI score (0–2) after the first dose of vaccine. A total of 104 (47.9%) participants were categorized as having a high AEFI score (5–20) and 113 (53.0%) as having a low AEFI score (0–4) after the second dose of vaccine. There were no significant differences in AEFI score by demographics, cancer characteristics, or current health condition for the first and second doses of the SARS-CoV-2 vaccine (Appendix 2).

**Figure 4:**
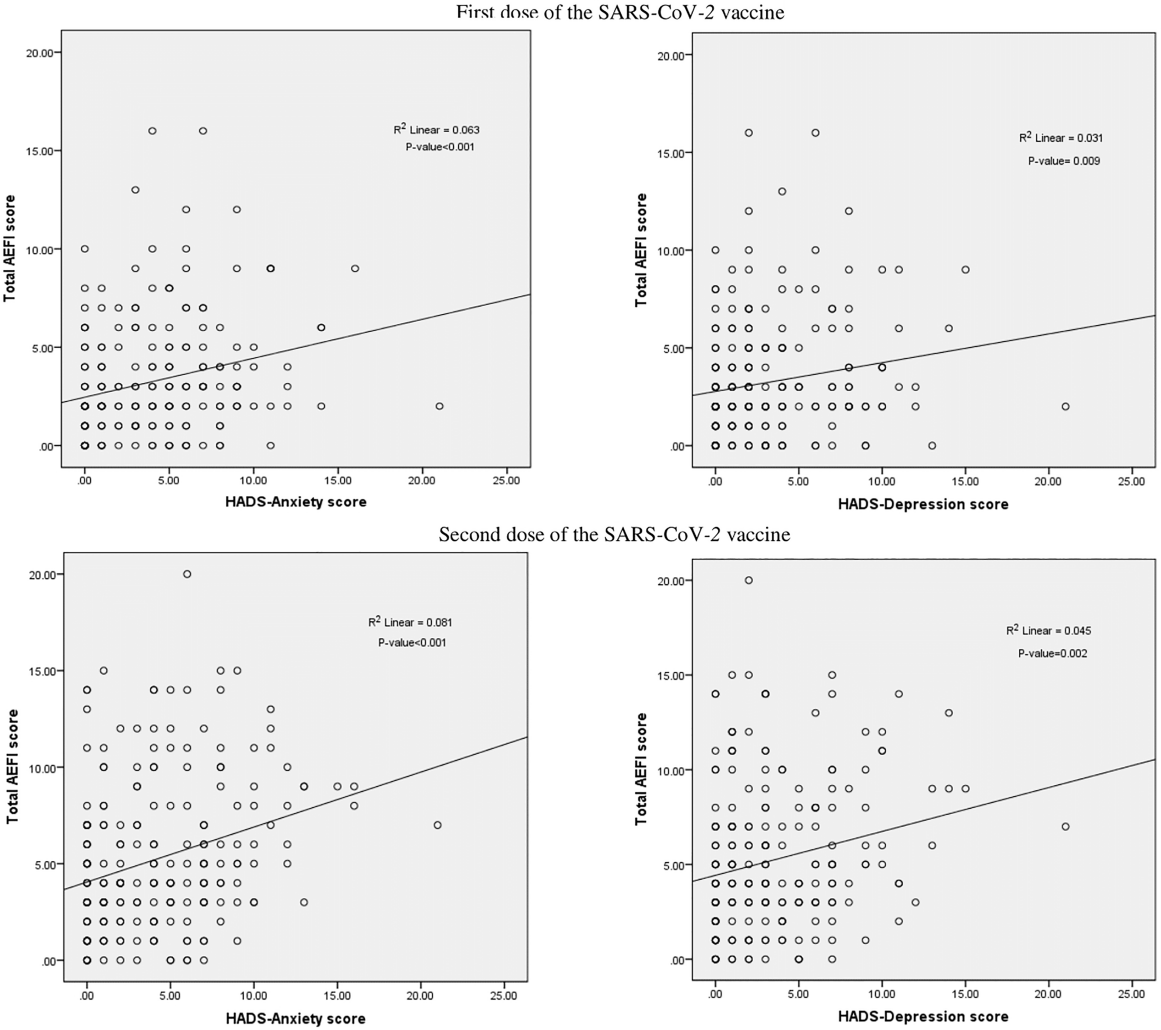
Correlation between total AEFI score and Hospital Anxiety and Depression Scale (HADS) score.

Figure 2 shows the associations between total AEFI and HADS scales. With regard to the first dose SARS-CoV-2 vaccine, positive correlations between the total AEFI and the HADS-A (*r* = 0.309, *p* < 0.001) and HADS-D scales (*r* = 0.214, *p* = 0.001) were observed. Similarly, positive associations were observed between the total AEFI and the HADS-A (*r* = 0.305, *p* < 0.001) and HADS-D (*r* = 0.235, *p* < 0.001) scales with regard to the second dose SARS-CoV-2 vaccine (Figure 4).

## Discussion

In Malaysia, similar to many countries around the world, as soon as the COVID-19 vaccine was available for the public, cancer patients/survivors were among the priority population groups for the COVID-19 vaccination program. This study evaluated the incidence and severity of AEFIs associated with the SARS-CoV-2 vaccines at a single COVID-19 vaccination center in a teaching university hospital during the early phase of the vaccination rollout and to provide a basis to ensure the safety of vaccination against SARS-CoV-2 among cancer patients/survivors. The psychological distress of patients before and after the first and second dose SARS-CoV-2 vaccine was also compared. We found that most participants had systemic and local AEFIs similar to those found in other studies reported in a systematic review (Chen et al., 2021). The severity of most AEFIs in this study was mild-to-moderate, and further levels of severity of AEFIs were higher with the second dose of the vaccine. There were also no serious events requiring hospitalization. Our results are consistent with a recent finding presented as conference abstracts that reported SARS-CoV-2 vaccines were well tolerated in patients diagnosed with cancer (Loew et al., 2021). Our results also may alleviate the concerns among cancer patients/survivors about adverse reactions of vaccination against SARS-CoV-2 and potentially contribute to improving the rate of inoculation in frail populations. We also found no significant differences in AEFIs by patients of various types of cancers and health conditions with both the first and second doses SARS-CoV-2 vaccines, implying that the safety of SARS-CoV-2 vaccines is reassuring to people affected by cancer. The finding of a higher severity of AEFIs with the second dose of vaccine when compared with the first dose of vaccine warrants further investigation. A systematic review reported no significant differences in AEFIs between the first and second doses of the vaccine (Chen et al., 2022).

During the COVID-19 pandemic, cancer patients/survivors around the world have experienced a range of dramatic disruptions in routine cancer care services and their everyday lives, resulting in a high prevalence of depression and anxiety symptoms. A previous local study on cancer patients/survivors sampled during the early phase of the COVID-19 pandemic in Malaysia reported high rates of symptoms of anxiety (29.0%) and depression (20.8%; Wong et al., 2021). In the current study, which is approximately 20 months into the pandemic, our baseline anxiety and depressive symptoms before receiving the SARS-CoV-2 vaccine were similar to those of the previous study in Malaysia during the early phase of the COVID-19 outbreak. This may imply that the depression and anxiety symptoms persist over time in cancer patients/survivors along the pandemic period before receiving the SARS-CoV-2 vaccine. Furthermore, this study also found that anxiety heightened among the patient/cancer survivors who were of poor health and with comorbidities. Hence, our findings suggest that cancer patients/survivors who are of poor health and with comorbidities should be provided counseling before vaccination, as they could be at increased risk for psychological distress.

The most important finding of this study is that the reduction in anxiety and depressive symptoms after receiving the first dose and reduction maintained throughout the second dose of the SARS-CoV-2 vaccine. Further lower rates of anxiety and depressive symptoms were reported after the second dose of the COVID-19 vaccine. A recent large-scale cross-sectional study similarly reported a significant reduction in anxiety and depressive symptoms after vaccination in U.S. adults (Chen et al., 2021). During the COVID-19 pandemic, patients with cancer are susceptible to psychological stress. As the rollout of the SARS-CoV-2 vaccines in Malaysia began during the period of rapid escalation of cases in the country, receiving the SARS-CoV-2 vaccine brought a new light and relief to cancer patients during the pandemic. As stress is a major precipitating psychological issue in patients from diagnosis through treatment and even after the disease is long gone (Kang et al., 2012), on a positive note, receiving the SARS-CoV-2 vaccine not only reduces their risk of infection but also alleviates their anxiety and depression. Furthermore, this also indicates the strong confidence in the SARS-CoV-2 vaccines within the communities of cancer patients/survivors.

To date, little is known about the association between the experience of AEFIs following SARS-CoV-2 vaccination and anxiety-related events in cancer patients/survivors. To the best of our knowledge, our study is among the first to provide evidence that a high severity of AEFIs was associated with higher anxiety and depressive symptoms. Patient-healthcare provider communication during SARS-CoV-2 vaccination is important to ease anxiety-related adverse events that might occur if patients experience AEFIs, especially owing to a growing body of misinformation surrounding the safety and effectiveness of the SARS-CoV-2 vaccines. A new channel for communication between healthcare providers and cancer patients/survivors should be established to provide the opportunity for patients to communicate with the healthcare team on various concerns after vaccination.

### Limitations

There are several limitations of this study that prevent the generalization of study results beyond the study sample population. First, the sample size is small and may not be representative of general cancer patient populations. Also, patients were recruited from one healthcare institution. The second limitation of our study stems from the cross-sectional design, which precludes concluding causal associations. It is also important to note that not all study participants received a similar type of vaccine. All study participants received the COVID-19 mRNA BNT162b2 vaccine, except two participants who received the Sinovac CoronaVac vaccine and one participant who received the Oxford-AstraZeneca vaccine. The participants who received the Sinovac CoronaVac and Oxford-AstraZeneca vaccines did not report higher serious adverse side effects than those generally experienced by those who received the COVID-19 mRNA BNT162b2 vaccine. It is also important to note that psychosocial and environmental factors such as family, social, and economic support, as well as pandemic-related stress that can potentially affect the emotion, anxiety, and depression in cancer patients (Taghadosi et al., 2017; Al-Quteimat and Amer, 2020), were unaccounted for, and possibly subtle confounding variables, and may have played a role in the outcome. Additionally, the comparison of perceived health status and psychological distress is suboptimal: perceived health is a subjective measure and hence prone to bias. Despite the above-stated limitations, our findings provide important insights into the relatively little empirical evidence of the psychological distress surrounding cancer patients/survivors receiving the SARS-CoV-2 vaccine.

## Conclusion

SARS-CoV-2 vaccination had a positive effect on decreasing the anxiety and depression levels of cancer patients/survivors, thus implying the benefit of vaccination in easing psychological distress during the COVID-19 pandemic. This study demonstrated the low risks and side effects of the SARS-CoV-2 vaccines for people affected by cancer. The findings of mild-to-moderate AEFIs found among cancer patients/survivors in this study help address vaccine hesitancy in cancer patients/survivors associated with concerns about severe adverse events associated with the SARS-CoV-2 vaccine. This study also uncovered that the experience of severe AEFIs may increase the risk of anxiety and depressive symptoms in people affected by cancer. Our findings suggest that healthcare providers should be made aware that people affected by cancer are vulnerable to an increase in psychological distress when experiencing the AEFIs of SARS-CoV-2 vaccination and provision of counseling to patients is important. An important implication of this study is that adverse psychological consequences of experiencing the AEFIs of SARS-CoV-2 vaccination should not be overlooked owing to the prominence of mental health conditions in patients diagnosed with cancer during the COVID-19 pandemic. Fear of receiving the SARS-CoV-2 vaccine contributes to enhancing emotional distress and together can increase vulnerability to long-lasting negative physical and psychological outcomes. Lastly, this study did not include other social environmental factors that may potentially influence the anxiety and depression levels of cancer patients/survivors during the vaccination period. Further research would be necessary to confirm these results and consider addressing a broad range of potential confounders.

## Data availability statement

The original contributions presented in the study are included in the article/Supplementary material, further inquiries can be directed to the corresponding authors.

## Ethics statement

The study was approved by the UMMC Medical Research Ethics Committee (MREC) (Approval code: MREC ID No: 202166-10200). The patients/participants provided their written informed consent to participate in this study.

## Author contributions

LW: conceptualization, formal analysis, methodology, project administration, supervision, software, writing—original draft, and writing—reviewing and editing. LL, MS, SS, CN, GH, TO, YW, PO, and JE: conceptualization, data curation, investigation, methodology, project administration, validation, and writing—reviewing and editing. HA: conceptualization, formal analysis, supervision, software, and writing—reviewing and editing. ZH: conceptualization, methodology, and writing—reviewing and editing. YL: conceptualization, methodology, funding acquisition, and writing—reviewing and editing. All authors contributed to the article and approved the submitted version.

## Funding

This work was supported by the Special Projects of the Central Government Guiding Local Science and Technology Development, China [No. 2021L3018]. The funder was not involved in study design, in the collection, analysis and interpretation of data; in the writing of the manuscript; nor in the decision to submit the manuscript for publication.

## Conflict of interest

The authors declare that the research was conducted in the absence of any commercial or financial relationships that could be construed as a potential conflict of interest.

## Publisher’s note

All claims expressed in this article are solely those of the authors and do not necessarily represent those of their affiliated organizations, or those of the publisher, the editors and the reviewers. Any product that may be evaluated in this article, or claim that may be made by its manufacturer, is not guaranteed or endorsed by the publisher.

## Supplementary material

The Supplementary materials for this article can be found online at: https://www.frontiersin.org/articles/10.3389/fpsyg.2022.906067/full#supplementary-material
