# Supplementary material for: Adverse events following immunization and psychological distress among cancer patients/survivors following vaccination against SARS-CoV-2 infection
Source: Front Psychol. 2022 Jul 26;13:906067. doi: 10.3389/fpsyg.2022.906067 (PMC9360916; doi:10.3389/fpsyg.2022.906067)
Supplement: Supplementary file 1 [file Data_Sheet_1.docx]

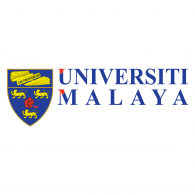


SIDE-EFFECTS AND PSYCHOLOGICAL WELL-BEING OF CANCER PATIENTS/SURVIVORS FOLLOWING COVID-19 VACCINATION

[PRE-VACCINATION]

**Section A: General Information**

| A1 | Age | _______________ years old |
| --- | --- | --- |
| A2 | Gender | [ ] Male  [ ] Female |
| A3 | Ethnicity | [ ] Malay  [ ] Chinese  [ ] Indian  [ ] Others |
| A4 | Religion | [ ] Islam  [ ] Buddhism  [ ] Hinduism  [ ] Christianity  [ ] Others |
| A5 | Highest educational level | [ ] No formal education  [ ] Primary  [ ] Secondary  [ ] College/University |
| A6 | Average monthly household income (MYR) | [ ] 5,000 and below  [ ] 5,001-10,000  [ ] More than10,000 |

**Section B: Cancer characteristics and health status**

| B1 | Number of cancer diagnosed with | [ ] 1  [ ] 2  [ ] More than 2 |
| --- | --- | --- |
| B2 | Duration of being diagnosed with cancer (years) |  |
| B3 | Stage of cancer in time of diagnosis | [ ] 0  [ ] 1  [ ] 2  [ ] 3  [ ] 4 |
| B4 | Do you have any chronic diseases other than cancer? | [ ] Yes  [ ] No |
| B5 | How do you rate your current health status | [ ] Poor  [ ] Fair  [ ] Good  [ ] Very good |

**Section C: Anxiety and Depression Assessment**

| AN1 | I feel tense or 'wound up | [ ] Most of the time  [ ] A lot of the time  [ ] From time to time, occasionally  [ ] Not at all |
| --- | --- | --- |
| DP1 | I still enjoy the things I used to enjoy | [ ] Definitely as much  [ ] Not quite so much  [ ] Only a little  [ ] Hardly at all |
| AN2 | I get a sort of frightened feeling as if  something awful is about to  happen | [ ] Very definitely and quite badly  [ ] Yes, but not too badly  [ ] A little, but it doesn’t worry me  [ ] Not at all |
| DP2 | I can laugh and see the funny side of things | [ ] As much as I always could  [ ] Not quite so much now  [ ] Definitely not so much now  [ ] Not at all |
| AN3 | Worrying thoughts go through my mind | [ ] A great deal of the time  [ ] A lot of the time  [ ] From time to time, but not too often  [ ] Only occasionally |
| DP3 | I feel cheerful | [ ] Not at all  [ ] Not often  [ ] Sometimes  [ ] Most of the time |
| AN4 | I can sit at ease and feel relaxed | [ ] Definitely  [ ] Usually  [ ] Not often  [ ] Not at all |
| DP4 | I feel as if I am slowed down | [ ] Nearly all the time  [ ] Very Often  [ ] Sometimes  [ ] Not at all |
| AN5 | I get a sort of frightened feeling like  'butterflies' in the stomach | [ ] Not at all  [ ] Occasionally  [ ] Quite Often  [ ] Very Often |
| DP5 | I have lost interest in my appearance | [ ] Definitely  [ ] I don't take as much care as I should  [ ] I may not take quite as much care  [ ] I take just as much care as ever |
| AN6 | I feel restless as I have to be on the  move | [ ] Very much indeed  [ ] Quite a lot  [ ] Not very much  [ ] Not at all |
| DP6 | I look forward with enjoyment to  things | [ ] As much as I ever did  [ ] Rather less than I used to  [ ] Definitely less than I used to  [ ] Hardly at all |
| AN7 | I get sudden feelings of panic | [ ] Very often indeed  [ ] Quite often  [ ] Not very often  [ ] Not at all |
| DP7 | I can enjoy a good book or radio or TV  program | [ ] Often  [ ] Sometimes  [ ] Not often  [ ]Very seldom |


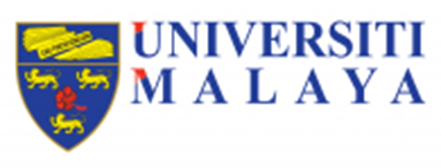


SIDE-EFFECTS AND PSYCHOLOGICAL WELL-BEING OF CANCER PATIENTS/SURVIVORS FOLLOWING COVID-19 VACCINATION

[1 WEEK AFTER FIRST DOSE VACCINE]

**Section A: Anxiety and Depression Assessment**

| AN1 | I feel tense or 'wound up | [ ] Most of the time  [ ] A lot of the time  [ ] From time to time, occasionally  [ ] Not at all |
| --- | --- | --- |
| DP1 | I still enjoy the things I used to enjoy | [ ] Definitely as much  [ ] Not quite so much  [ ] Only a little  [ ] Hardly at all |
| AN2 | I get a sort of frightened feeling as if  something awful is about to  happen | [ ] Very definitely and quite badly  [ ] Yes, but not too badly  [ ] A little, but it doesn’t worry me  [ ] Not at all |
| DP2 | I can laugh and see the funny side of things | [ ] As much as I always could  [ ] Not quite so much now  [ ] Definitely not so much now  [ ] Not at all |
| AN3 | Worrying thoughts go through my mind | [ ] A great deal of the time  [ ] A lot of the time  [ ] From time to time, but not too often  [ ] Only occasionally |
| DP3 | I feel cheerful | [ ] Not at all  [ ] Not often  [ ] Sometimes  [ ] Most of the time |
| AN4 | I can sit at ease and feel relaxed | [ ] Definitely  [ ] Usually  [ ] Not often  [ ] Not at all |
| DP4 | I feel as if I am slowed down | [ ] Nearly all the time  [ ] Very Often  [ ] Sometimes  [ ] Not at all |
| AN5 | I get a sort of frightened feeling like  'butterflies' in the stomach | [ ] Not at all  [ ] Occasionally  [ ] Quite Often  [ ] Very Often |
| DP5 | I have lost interest in my appearance | [ ] Definitely  [ ] I don't take as much care as I should  [ ] I may not take quite as much care  [ ] I take just as much care as ever |
| AN6 | I feel restless as I have to be on the  move | [ ] Very much indeed  [ ] Quite a lot  [ ] Not very much  [ ] Not at all |
| DP6 | I look forward with enjoyment to  things | [ ] As much as I ever did  [ ] Rather less than I used to  [ ] Definitely less than I used to  [ ] Hardly at all |
| AN7 | I get sudden feelings of panic | [ ] Very often indeed  [ ] Quite often  [ ] Not very often  [ ] Not at all |
| DP7 | I can enjoy a good book or radio or TV  program | [ ] Often  [ ] Sometimes  [ ] Not often  [ ]Very seldom |

**Section B: Side effect following first dose vaccination**

| B1 | Pain/ swelling/redness of the injection site | [ ] None  [ ] Mild  [ ] Moderate  [ ] Severe |
| --- | --- | --- |
| B2 | Tiredness | [ ] None  [ ] Mild  [ ] Moderate  [ ] Severe |
| B3 | Headache | [ ] None  [ ] Mild  [ ] Moderate  [ ] Severe |
| B4 | Chills | [ ] None  [ ] Mild  [ ] Moderate  [ ] Severe |
| B5 | Joint pain | [ ] None  [ ] Mild  [ ] Moderate  [ ] Severe |
| B6 | Fever | [ ] None  [ ] Mild  [ ] Moderate  [ ] Severe |
| B7 | Nausea | [ ] None  [ ] Mild  [ ] Moderate  [ ] Severe |
| B8 | Feeling unwell | [ ] None  [ ] Mild  [ ] Moderate  [ ] Severe |
| B9 | Swelling at the lymph node | [ ] None  [ ] Mild  [ ] Moderate  [ ] Severe |
| B10 | Blood clot/thrombosis event | [ ] None  [ ] Mild  [ ] Moderate  [ ] Severe |
| B11 | Others | ____________________________________ |

\


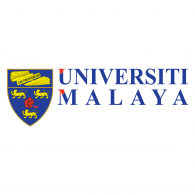


SIDE-EFFECTS AND PSYCHOLOGICAL WELL-BEING OF CANCER PATIENTS/SURVIVORS FOLLOWING COVID-19 VACCINATION

[1 WEEK AFTER SECOND DOSE VACCINE]

**Section A: Anxiety and Depression Assessment**

| AN1 | I feel tense or 'wound up | [ ] Most of the time  [ ] A lot of the time  [ ] From time to time, occasionally  [ ] Not at all |
| --- | --- | --- |
| DP1 | I still enjoy the things I used to enjoy | [ ] Definitely as much  [ ] Not quite so much  [ ] Only a little  [ ] Hardly at all |
| AN2 | I get a sort of frightened feeling as if  something awful is about to  happen | [ ] Very definitely and quite badly  [ ] Yes, but not too badly  [ ] A little, but it doesn’t worry me  [ ] Not at all |
| DP2 | I can laugh and see the funny side of things | [ ] As much as I always could  [ ] Not quite so much now  [ ] Definitely not so much now  [ ] Not at all |
| AN3 | Worrying thoughts go through my mind | [ ] A great deal of the time  [ ] A lot of the time  [ ] From time to time, but not too often  [ ] Only occasionally |
| DP3 | I feel cheerful | [ ] Not at all  [ ] Not often  [ ] Sometimes  [ ] Most of the time |
| AN4 | I can sit at ease and feel relaxed | [ ] Definitely  [ ] Usually  [ ] Not often  [ ] Not at all |
| DP4 | I feel as if I am slowed down | [ ] Nearly all the time  [ ] Very Often  [ ] Sometimes  [ ] Not at all |
| AN5 | I get a sort of frightened feeling like  'butterflies' in the stomach | [ ] Not at all  [ ] Occasionally  [ ] Quite Often  [ ] Very Often |
| DP5 | I have lost interest in my appearance | [ ] Definitely  [ ] I don't take as much care as I should  [ ] I may not take quite as much care  [ ] I take just as much care as ever |
| AN6 | I feel restless as I have to be on the  move | [ ] Very much indeed  [ ] Quite a lot  [ ] Not very much  [ ] Not at all |
| DP6 | I look forward with enjoyment to  things | [ ] As much as I ever did  [ ] Rather less than I used to  [ ] Definitely less than I used to  [ ] Hardly at all |
| AN7 | I get sudden feelings of panic | [ ] Very often indeed  [ ] Quite often  [ ] Not very often  [ ] Not at all |
| DP7 | I can enjoy a good book or radio or TV  program | [ ] Often  [ ] Sometimes  [ ] Not often  [ ]Very seldom |

**Section B: Side effect following first dose vaccination**

| B1 | Pain/ swelling/redness of the injection site | [ ] None  [ ] Mild  [ ] Moderate  [ ] Severe |
| --- | --- | --- |
| B2 | Tiredness | [ ] None  [ ] Mild  [ ] Moderate  [ ] Severe |
| B3 | Headache | [ ] None  [ ] Mild  [ ] Moderate  [ ] Severe |
| B4 | Chills | [ ] None  [ ] Mild  [ ] Moderate  [ ] Severe |
| B5 | Joint pain | [ ] None  [ ] Mild  [ ] Moderate  [ ] Severe |
| B6 | Fever | [ ] None  [ ] Mild  [ ] Moderate  [ ] Severe |
| B7 | Nausea | [ ] None  [ ] Mild  [ ] Moderate  [ ] Severe |
| B8 | Feeling unwell | [ ] None  [ ] Mild  [ ] Moderate  [ ] Severe |
| B9 | Swelling at the lymph node | [ ] None  [ ] Mild  [ ] Moderate  [ ] Severe |
| B10 | Blood clot/thrombosis event | [ ] None  [ ] Mild  [ ] Moderate  [ ] Severe |
| B11 | Others | ____________________________________ |
