# Supplementary material for: Adverse events following immunization and psychological distress among cancer patients/survivors following vaccination against SARS-CoV-2 infection
Source: Front Psychol. 2022 Jul 26;13:906067. doi: 10.3389/fpsyg.2022.906067 (PMC9360916; doi:10.3389/fpsyg.2022.906067)
Supplement: Supplementary file 2 [file Data_Sheet_2.docx]

**Appendix 2** Factors associated with adverse events following immunization (AEFIs) score after first dose and second dose of the SARS-CoV-2 vaccine

|  | Univariable analysis | | Multivariable analysis |  | Univariable analysis | |
| --- | --- | --- | --- | --- | --- | --- |
|  | First-dose | |  |  | Second-dose | |
| *Socio-demography* | High side effect score  (3-16)  (n=102) | p-value | OR (95 CI%)  (3-16 *vs* 0-2) |  | High side effect score  (5-20)  (n=104) | p-value |
| Age group (years) |  |  |  |  |  |  |
| 19-40 | 30 (57.7) | 0.044 | 2.03 (0.98-4.21) |  | 27 (51.9) | 0.381 |
| 41-50 | 41 (50.6) |  | 1.57 (0.83-2.96) |  | 42 (51.9) |  |
| 60-81 | 31 (36.9) |  | Reference |  | 35 (42.2) |  |
| Gender |  |  |  |  |  |  |
| Male | 16 (47.1) | 1.000 |  |  | 17 (50.0) | 0.853 |
| Female | 86 (47.0) |  |  |  | 87 (47.8) |  |
| Ethnicity |  |  |  |  |  |  |
| Malay | 14 (35.0) | 0.204 |  |  | 19 (47.5) | 0.227 |
| Chinese | 75 (48.4) |  |  |  | 71 (46.1) |  |
| Indian | 6 (50.0) |  |  |  | 6 (50.0) |  |
| Others | 7 (70.0) |  |  |  | 8 (80.0) |  |
| Religion |  |  |  |  |  |  |
| Islam | 14 (32.6) | 0.054 |  |  | 19 (44.2) | 0.959 |
| Buddhism | 53 (47.7) |  |  |  | 53 (48.2) |  |
| Hinduism | 6 (54.5) |  |  |  | 6 (54.5) |  |
| Christianity | 20 (48.8) |  |  |  | 20 (48.8) |  |
| Others | 9 (81.8) |  |  |  | 6 (54.5) |  |
| Highest educational level |  |  |  |  |  |  |
| Secondary and below | 23 (35.4) | 0.027 | Reference |  | 25 (39.1) | 0.101 |
| Tertiary | 79 (52.0) |  | 1.70 (0.91-3.16) |  | 79 (52.0) |  |
| Average monthly household income (MYR) |  |  |  |  |  |  |
| ≤5,000 | 49 (43.0) | 0.266 |  |  | 54 (47.8) | 0.815 |
| 5,001-10,000 | 37 (55.2) |  |  |  | 31 (46.3) |  |
| > 10,000 | 16 (44.4) |  |  |  | 19 (52.8) |  |
| *Cancer characteristics* |  |  |  |  |  |  |
| Number of cancer diagnosed with |  |  |  |  |  |  |
| 1 | 91 (46.2) | 0.488 |  |  | 93 (47.4) | 0.640 |
| >1 | 11 (55.0) |  |  |  | 11 (55.0) |  |
| Duration of being diagnosed with cancer (years) |  |  |  |  |  |  |
| < 1 | 5 (33.3) | 0.447 |  |  | 9 (64.3) | 0.239 |
| 1-5 | 74 (49.3) |  |  |  | 67 (44.7) |  |
| >5 | 23 (44.2) |  |  |  | 28 (53.8) |  |
| Level of cancer diagnosed with |  |  |  |  |  |  |
| 0 | 12 (48.0) | 0.261 |  |  | 10 (40.0) | 0.004 |
| I | 25 (43.9) |  |  |  | 35 (61.4) |  |
| II | 40 (57.1) |  |  |  | 35 (50.0) |  |
| III | 16 (36.4) |  |  |  | 11 (25.6) |  |
| IV | 9 (42.9) |  |  |  | 13 (61.9) |  |
| *Current health condition* |  |  |  |  |  |  |
| Diagnosed with any comorbidities |  |  |  |  |  |  |
| Yes | 18 (60.0) | 0.167 |  |  | 17 (56.7) | 0.332 |
| No | 84 (44.9) |  |  |  | 87 (46.8) |  |
| Perceived current health status |  |  |  |  |  |  |
| Very good/Good | 59 (43.4) | 0.206 |  |  | 67 (49.3) | 0.675 |
| Fair/Poor | 43 (53.1) |  |  |  | 37 (46.2) |  |

Hosmer–Lemeshow test, chi-square: 0.990, *p*-value: 0.911; Nagelkerke R^2^ : 0.055
